# Social spatial structure and psychological mechanisms of seat-occupying behavior in university libraries—based on grounded theory analysis

**DOI:** 10.3389/fpsyg.2026.1863222

**Published:** 2026-10-07

**Authors:** Xiuli Li, Jiaojiao Cheng

**Affiliations:** Jilin Business and Technology College, Changchun, China

**Keywords:** alienation of control rights, environmental psychology, grounded theory, library seat occupation behavior, spatial control rights, territoriality, university students

## Abstract

Occupying seats in university libraries is a situated practice shaped by the interaction of resource availability, environmental-psychological needs, institutional rules, and student responses under academic pressure. To reveal the underlying social logic of its emergence, grounded theory was employed to conduct coding analysis on 496 analyzable units derived from heterogeneous documentary, public-discourse, visual, and observational sources, and a multidimensional theoretical model of the social spatial structure of seat occupancy in university libraries was constructed. The analysis suggests that the limited availability of seating resources and students’ psychological needs for spatial stability, personal space, and privacy may jointly contribute to seat-occupying practices, while design features, task requirements, and individual preferences may shape which seats become desirable. In the complex governance of management and technology, students may adopt strategic, affective, and territorial responses that can be reinforced through feedback processes and may contribute to the alienation of spatial functions and the dissipation of public resources, which the proposed interpretive model summarizes as a possible process of “resource scarcity-power conflict-spatial alienation” and feedback. The construction of this model aims to provide theoretical references for libraries to optimize psychologically informed management practices, curb seat occupying behavior, and enhance resource utilization efficiency.

## 1 Introduction

With the continuous deepening of the popularization of higher education, the contradictions in the allocation of resources in university libraries, as core academic spaces, are becoming increasingly prominent. Although the Ministry of Education’s “Regulations for Libraries of General Colleges and Universities” (Ministry of Education of the People's Republic of China, 2015) explicitly requires “optimizing the allocation of space resources and ensuring the fair use rights of readers,” colleges and universities have successively implemented measures such as seat reservation systems and timed clearing of seats, attempting to alleviate the conflict of seat occupation. However, in actual operation, such technocratic and disciplinary governance approaches are still difficult to fundamentally respond to the complex social spatial interaction mechanisms behind the phenomenon of seat occupation. Existing studies and public discussions continue to report practical difficulty in accessing seats, suggesting a gap between nominal resource provision and effective use. This phenomenon suggests that the current understanding of seat-occupying behavior mostly remains at the superficial level of resource supply and demand imbalance, or tends to appeal to moral norms, and has not yet fully penetrated into the spatial practice logic behind the behavior, the strategic responses of student groups, and the interactive structure of space-power relations. From an economic perspective, Sun and Yu (2010), and Hardin (1968) have started from “resource competition” and revealed the conflict between individual rationality and collective interests. However, they simplify the seat as a homogeneous, disputable object, ignoring the social significance of space itself and the complexity of student behavior. Gayton (2008) distinguishes the communal character of academic libraries from a purely social model and emphasizes the role of shared norms in supporting focused study. This perspective suggests that seat use should be examined in relation to the collectively maintained purposes of library space rather than reduced to individual morality. Applied seat-management research (Liu et al., 2021) demonstrates the practical value of mobile reservation systems, whereas Suchman’s situated-action perspective (Suchman, 1987) cautions that the operation of a technical rule depends on how users interpret and enact it in specific settings. The above perspectives tend to abstract the seat occupation behavior from specific social and spatial contexts, viewing it as a static phenomenon driven by several isolated variables, and thus have not fully captured the generation and evolution mechanism of behavior in dynamic interaction. Existing research often starts from a single dimension, emphasizing resource competition, focusing on psychological motivation, or emphasizing technical control, lacking a systematic analytical framework that can integrate space, subjects, and the practice process. Especially when explaining how students form and maintain the “spontaneous order” of seat occupation in continuous interaction with management systems, spatial layout, and other readers, their explanatory power seems limited.

Therefore, to compensate for the deficiencies in the connection between micro-individual behavior and macro spatial structure in existing research, this study introduces a social spatial perspective and adopts the grounded theory as a qualitative research method. It systematically develops concepts and categories from source materials concerning seat-occupying behavior in university libraries and constructs an interpretive model grounded in the analyzed settings. The study asks how resource conditions, designed spatial features, institutional practices, and student responses interact in accounts of university-library seat occupation, and how these relationships can be organized through open, axial, and selective coding. The resulting framework traces four linked analytical stages—conditions, intervention, interaction, and feedback—without treating these links as statistically established causal effects. Ultimately, the research aims to provide a theoretical reference for constructing a library space governance path that integrates fairness, efficiency, and humanistic care.

## 2 Literature review

Currently, the research on university library is experiencing a profound paradigm shift, and the academic community is gradually moving beyond the traditional analytical path of “resources-technology-space” and shifting to reconstructing the understanding of library space from the dimension of social relationships. This shift resonates with the rise of new space concepts such as “learning shared spaces” and “maker spaces,” jointly promoting the cognitive paradigm evolution of the library from a functional carrier to a “field of social relationship reproduction.” Existing research mainly focuses on two aspects: the behavior of occupying spaces in university libraries and social space theory.

### 2.1 Occupation behavior in university libraries

#### 2.1.1 Factors influencing occupancy behavior

Studies of library layout and use have linked seat choice to the number and distribution of seats, proximity to windows and power outlets, visibility, and circulation patterns (Nonci et al., 2026; Fan et al., 2022). Research on spatial configuration likewise indicates that visual accessibility, zoning, and the relationship between circulation and seating may affect how particular locations are used (Hillier and Hanson, 1984). These design-related influences should be considered alongside overall seat availability rather than interpreted as evidence of a single competition mechanism.

#### 2.1.2 Governance practices of occupancy behavior

Research in this area examines rule design, enforcement, and the use of reservation or occupancy-monitoring technologies. Prototype studies show that real-time occupancy detection and reservation systems can improve information transparency and support seat allocation (El-Khouly et al., 2026; Maepa and Moeti, 2021). At the same time, the technical literature identifies unresolved issues of implementation, usability, and circumvention. A systematic review of smart seat-occupancy and reservation systems summarizes both their operational benefits and their persistent technical and governance limitations (Reneilwe Maepa and Nthabiseng Moeti, 2024). A recent governance-oriented reservation study likewise emphasizes transparent rules, check-in validation, automated release of unused bookings, and administrative oversight (Rana et al., 2026). Together, these studies indicate that technical design and governance rules should be assessed jointly, while their effects remain dependent on institutional and user contexts.

#### 2.1.3 Environmental-psychological and symbolic logic of occupancy behavior

Researchers have begun to pay attention to the symbolic significance behind seat-taking behavior. Recent work conceptualizes the university library as a spatial assemblage in which campus, collections, and student practices intersect (Leorke et al., 2026). Research on atmosphere and affordance also helps explain why environmental qualities can acquire emotional and motivational significance for users (Arbib, 2021). Environmental-psychology research on personal space further shows that spatial arrangement, privacy needs, and territorial cues can shape how users interpret access and proximity (Sommer, 1969). Studies of the university library as a place of possibility likewise stress that users actively reinterpret spaces rather than simply occupy predetermined functions (O'Donnell and Anderson, 2022). These perspectives support examining personal items and repeated use as possible territorial signals, without inferring psychological ownership from observation alone.

### 2.2 Social space theory

#### 2.2.1 The origins and core connotations of social space theory

Social space theory emerged in the middle and late 20th century with the trend of “spatial turn,” with its core breakthrough being the redefinition of space from a static material container to a dynamic field with social productivity and practicality. The theory emphasizes that space is not a neutral background but a product of social relations, and it also mediates and reshapes social relations in return. The French thinker Henri Lefebvre, as the founder, proposed the “triple dialectic of space” in “The Production of Space,” systematically explaining the dialectical relationship among spatial practice, the representation of space, and the space of representation (Lefebvre, 1991). On this basis, David Harvey proposed the concept of “spatial–temporal compression” from the logic of capital accumulation, revealing how capital overcomes geographical barriers through accelerated flow and further criticizes the lack of justice and the mechanism of unequal reproduction contained in spatial production (Harvey, 1989). Overall, social space theory views space as a field full of power interactions and dynamic reproduction, providing a key theoretical perspective for in-depth analysis of social behavior.

#### 2.2.2 The compatibility between social space theory and occupation behavior

Social space theory provides an analytical perspective for considering the library as both a designed physical setting and a social setting in which rules, meanings, and interactions are negotiated. From this perspective, occupying seats is essentially a form of spatial practice, where students not only gain learning positions but also participate in the dynamic construction of the library’s social space. Within this study, power, inequality, and resistance are used as sensitizing concepts rather than predetermined findings; competition is treated as a possible mechanism under actual or anticipated scarcity. At the same time, social space theory’s emphasis on the co-production of space and society offers a potentially relevant lens for grounded-theory analysis of process and interaction. This combined perspective also leaves room for complementary explanations involving layout, zoning, lighting, acoustics, equipment, privacy, task requirements, and individual preferences, all of which may influence which seats become scarce or desirable.

Existing research often analyzes the behavior of occupying seats in university libraries from a single perspective, such as economics, psychology, or management, lacking an integrated framework of space, subject, and practice. It is difficult to deeply explain the formation and maintenance mechanism of seat occupation as a “spontaneous order” in continuous interaction. At the same time, such research often abstracts the behavior of occupying seats from specific social space contexts, viewing it as a static, isolated variable-driven phenomenon, ignoring its evolution logic and interaction complexity as a dynamic spatial practice. To make up for the above deficiencies, this study adopts the grounded theory method, obtains original data such as structured observation and relevant documents through purposive sampling, focuses on the multiple perspectives of seat occupiers, anti-occupiers, and managers, and follows the three-level procedures of open coding, axial coding, and selective coding to construct a social space structure theoretical model of seat occupation behavior. The remaining materials within the predefined corpus were then examined through constant comparison to refine the framework and assess whether additional categories or relationships emerged; the resulting model is presented as an interpretive account within the studied corpus.

## 3 Research design

### 3.1 Research method

Grounded theory is a systematic qualitative research method (Glaser and Strauss, 1967). Its core lies in directly constructing a theoretical framework from empirical data, rather than presupposing hypotheses or relying on quantitative analysis. This method emphasizes the detailed collection and analysis of original data, using inductive logic, to refine concepts and categories from the bottom up, and gradually develop theoretical interpretations of the phenomena under study (Glaser and Strauss, 1967). Grounded theory is particularly concerned with the exploration and interpretation of meaning, adept at analyzing the complex relationships between interpersonal interaction processes and individual behavior and social structures, and has both theoretical creation and development functions. Although existing research has already analyzed occupying behavior from the perspectives of resources, psychology, and management, there is still a lack of systematic theoretical construction. The path of grounded theory “building theory from experience” justly fills this gap. In view of the focus of this study on the social spatial structure behind the occupying behavior in university libraries, this behavior has significant hidden and highly contextualized characteristics, involving individual strategies, spatial physical configuration, rule negotiation, and dynamic interactions among multiple parties. The grounded theory method shows significant relevance to this issue, mainly because it can start from the specific interactions and contexts of occupying behavior and refine concepts from the bottom up. This method is good at analyzing the dynamic interaction processes of individual strategies, spatial configuration, and rule negotiation, and can effectively reveal the hidden and highly contextualized social spatial structure behind the occupying phenomenon. The specific research flowchart is detailed in Figure 1.

**Figure 1:**
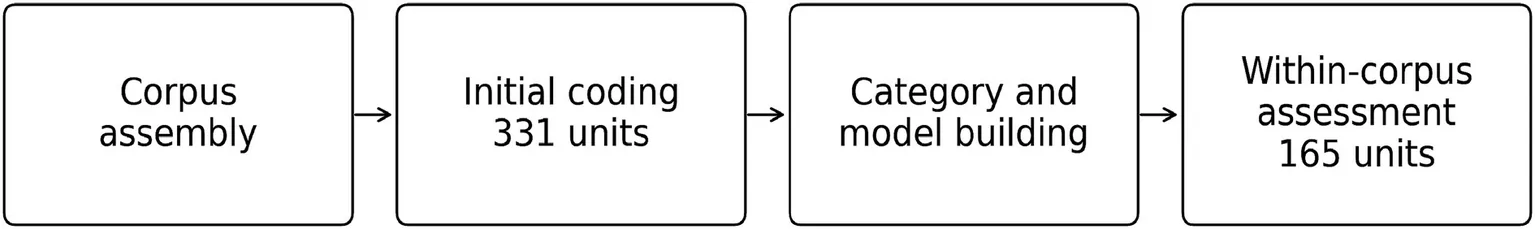
Flowchart of data collection and analysis (Source: Authors’ analysis). The final step assesses additional categories or relationships within the retained corpus; it is not independent theoretical verification.

### 3.2 Sample selection and data collection

To deeply explore the social spatial structure behind the seating behavior in university libraries through grounded theory, this study systematically collected three broad types of source material. A source item refers to an original document, post, image, log entry, or observation record, whereas an analyzable unit refers to a discrete excerpt or record entered into coding; source counts and coded units are therefore reported separately. The first type comprises contextual documentary materials, including policy documents, institutional records, academic publications, and relevant visual materials. These established texts and archives were used to provide background and institutional context; where discrete excerpts were entered into coding, they were treated as documentary evidence rather than as direct observations of student behavior. Specifically, 8 policy documents on seat management were collected from the official website of the Ministry of Education and the websites of various university libraries; 40 academic publications were consulted through databases such as CNKI, Web of Science, and SpringerLink using keywords such as “seating in university libraries” and “space governance,” with a publication window of 2019–2024. The 2019–2024 window was selected to capture recent discussion of social space, space production, smart-library technologies, and changes in library use surrounding the COVID-19 pandemic. The bounded period provides a contemporary contextual frame; it refers to the publication dates of these materials, not to the dates of social-media retrieval or field observation. Of the 40 academic publications, 23 contributed 52 discrete excerpts to the coded corpus, whereas the remaining 17 were used only as background literature. An itemized inventory identifying these 23 publications and all 40 consulted publications is not available for reporting, which limits source-level traceability. In addition, internal documents such as library annual reports and spatial layout maps, together with visual materials, were used to characterize institutional and spatial contexts. The second type is public opinion materials, covering news media reports and open data from social media. These materials have broad public visibility and temporal immediacy, helping to reflect contemporaneous public attention to and discussion of seat-occupying behavior. Specifically, 10 related news reports published during 2019–2024 were collected from important newspaper full-text databases, China News Network, Huasheng Online, and other media platforms; and approximately 200 publicly accessible posts and comments that directly discussed university-library seating were collected. The source descriptions identify Zhihu, Weibo, university forums, Xiaohongshu, Douyin, Bilibili, and WeChat public accounts. An item-level platform breakdown is not available. The exact retrieval dates, social-media search strings, collection procedure, duplicate-handling procedure, and reviewer-level screening decisions cannot be reconstructed from the documentation available for reporting. The literature-search keywords given above should therefore not be interpreted as a documented social-media search strategy. These materials were analyzed as records of publicly expressed discourse, not as direct evidence of users’ unobserved intentions or mental states. The third type is empirical materials, including structured observation records and typical cases. These materials provided contextual evidence derived from practical settings and complemented the documentary and public-discourse sources. Specifically, structured observation comprised an aggregate of 30 days and 60 h across three university libraries, together with three records of technical interventions. The observations were represented in observation records/logs, from which visible practices were entered into coding. The available documentation does not specify the allocation of days and hours to each library, the observation dates, the recording template, or whether entries were made contemporaneously or subsequently. Accordingly, these totals are reported only for the three libraries combined, without assuming equal observation time at each site. During the observation period, Library A operated a comprehensive seat-reservation system, with time-limited release rules for temporarily vacated seats, scheduled seat-clearing procedures, and frequent staff patrols. Library B combined partial reservation with on-site seat selection, implemented periodic seat-clearing procedures, and conducted less frequent staff patrols. Library C did not use a seat-reservation system and relied mainly on a student seat-use convention and occasional manual clearing interventions. The observations were used to identify recurring visible practices within these settings rather than to estimate prevalence or establish causal differences between institutions. Together, the complementary source types provide a contextual and empirical basis for the analysis of social spatial structure.

This study selected the three broad types of source material based on the following considerations: First, the combination of documentary, public-discourse, and observational materials supports comparison across source types. Second, contextual documents address institutional and normative settings, public materials present expressed discourse, and observations record visible practices, together covering physical configuration, rule operation, and situated negotiation. Finally, through comprehensive comparison and analysis, it can more systematically reveal the tacit logic and dynamic evolution mechanism behind the occupation behavior. In the research process, guided by relevance to the research question and constant comparison within the predefined corpus, this study uses whether it directly reflects “spatial practice, rights conflict, and institutional interaction” as the core criterion, and conducts a second screening of all collected data to ensure that the retained materials are closely centered around the core issue of “the generation mechanism of social spatial structure” and provide an effective analytical basis for the interconstructive mechanism of behavior, institutions, and space. After relevance screening and segmentation of the heterogeneous source materials, 496 analyzable units were retained for coding: 24 policy-document units, 18 news-media units, 52 academic-publication units, 120 social-media units, 36 physical-document units, 30 visual-material units, 160 observation units, 40 backend-log units, 8 technical-intervention units, and 8 case units. These units were discrete textual excerpts, visual records, log entries, or observation records rather than 496 source files. An initial set of 331 analyzable units was coded iteratively, comprising 16 policy, 12 news, 35 academic, 80 social-media, 24 physical-document, 20 visual, 107 observation, 27 backend-log, 5 technical-intervention, and 5 case units. The remaining 165 units comprised 8, 6, 17, 40, 12, 10, 53, 13, 3, and 3 units from the same source types, respectively, and were subsequently examined through constant comparison to assess whether new concepts, category properties, or relationships emerged. This was a sequential assessment within a predefined corpus, not a statistical training-validation split or continued theoretical sampling. The preliminary category system had stabilized after the initial 331 units; the remaining 165 units were examined for additional categories or relationships within that same corpus (Section 4.5). The original source items and the composition of the analyzable units are reported in Table 1; the original collection quantities differ by data type and should not be summed as a single sample total.

**Table 1:** Profile of data sources, collection units, and analyzable units.

Serial number	Data type	Source	Quantity
1	Policy Document	Ministry of Education “Regulations for Libraries of Regular Colleges and Universities”	3 copies
		Higher Education Library Autonomous Regulations (University-level)	5 copies; 24 analyzable units (16 initial; 8 subsequent assessment)
2	News Media	Mainstream Media (China News Network https://www.chinanews.com.cn/)	5 articles
		Local Media (Huasheng Online/https://www.voc.com.cn/)	5 articles; 18 analyzable units (12 initial; 6 subsequent assessment)
3	Contextual Literature	CNKI/Web of Science Journal Papers	30 journal articles
		Doctoral/PhD Thesis	10 theses; 52 analyzable units from 23 publications (35 initial; 17 subsequent assessment); 17 publications used as background only
4	Social Media Data	University BBS/Zhihu/Xiaohongshu/Weibo Topic Discussions	200 posts; 120 analyzable units (80 initial; 40 subsequent assessment)
		Short Video Platforms (Douyin/Bilibili)	
		WeChat Official Account (Student Works/In-depth Articles)	
5	Physical Document	Library Seat Management System Document	12 copies
		Student Seat Occupation Agreement/Forum Discussion Record	15 copies; 36 analyzable units across 27 documents (24 initial; 12 subsequent assessment)
		Seat Reservation System Backend Log	2 months; 40 analyzable units (27 initial; 13 subsequent assessment)
6	Visual Materials	Seat-taking item type and space marking photo	100 sheets
		Library space zoning and seat vacancy rate heatmap	5 sheets; 30 analyzable units across 105 visual records (20 initial; 10 subsequent assessment)
7	Structured Observation	Peak hour (opening hours/examination season) seat competition behavior	60 h; 160 analyzable units (107 initial; 53 subsequent assessment)
		Technical Intervention Process (Scanning System Use, Seat Occupation Cleaning)	3 records; 8 analyzable units (5 initial; 3 subsequent assessment)
8	Real Case	Baidu Tieba	3 cases; 8 analyzable units (5 initial; 3 subsequent assessment)

Source: Authors’ analysis.

## 4 Category refinement and model construction

### 4.1 Open-ended coding extraction of concepts and categories

Open coding is the starting point of grounded theory analysis, its core lies in the careful reading and marking of the original materials line by line and sentence by sentence, with the aim of identifying preliminary conceptual prototypes and gradually refining more abstract categories (Glaser and Strauss, 1967). In this study, the qualitative analysis software Nvivo 14.0 was used to process the various types of text materials collected. First, the materials were organized according to the year and type of publication, and then all original statement fragments related to the phenomenon of occupying seats in university libraries were selected and extracted, and conceptual coding was assigned to these fragments. For example, when seeing students “writing ‘This seat is occupied, please do not sit here’ on a sticky note” or “laying out a ‘warning line’ on the ground with an AirPods case and a data cable,” such specific behaviors are abstractly generalized as the concept of ‘symbolic territory.’ This stage identified 64 initial concepts. For reporting, labels beginning with a denote representative source excerpts, b denotes initial concepts, A denotes initial categories, and B denotes main categories, allowing the progression from evidence to concepts and categories to be followed across Tables 2, 3. The source excerpts in Table 2 retain the wording of the underlying accounts, including causal or evaluative expressions; these represent source perspectives rather than causal conclusions established by this study. Seven excerpts in Table 2 are accompanied by source-type and internal source identifiers to illustrate traceability; these examples do not constitute a complete coding matrix. The internal source codes used in these examples are P (policy), N (news), S (social media), O (observation), and L (backend log). Next, a deep-level abstraction process was conducted, classifying them into broader categories. This process requires a careful examination of the core connotation of each concept and its association with other concepts. Concepts such as “rule flexibility,” “clearing rules,” and “rule resistance,” although with different specific manifestations, all revolve around the various rules and measures formulated by the library to manage the behavior of occupying seats and the reactions they trigger. Therefore, they are merged and integrated into a higher-level category, refined into “the duality of institutional rules.” Through such a step-by-step analytical process, a total of 22 initial categories were finally refined. See Table 2 for details.

**Table 2:** Open coding table.

Initial category	Concept coding	Original statement
A7 Space Order Game	b1 Space discipline	a1 The high school library should reasonably allocate reading seats, establish a seat reservation system, maintain public order, and prevent uncivilized phenomena such as occupying seats.
	b2 Body Discipline	a2 at 4:30 a.m., a 100-meter long queue at the library entrance, occupying seats has evolved into a ritual of “being physically present.” [Source type: News media; source ID: N-04-01.]
	b3 Space Purification	a3 Volunteers clean up the desktop clutter at 10 p.m. every night, store it by date, and dispose of expired items.
A15 Symbolic Sovereignty Spatial Construction	b4 Symbol territory	a4 write “This seat is occupied, do not sit” on a sticky note; arrange the AirPods case and data cable into a “caution line.” [Source type: Social media; source ID: S-058-01.]
	b5 Item sovereignty	a5 (the poster): “Early morning 8 o’clock rush to open, one book + one power bank = a day’s seat sovereignty”;
	b6 Sticky Note Sovereignty	a6 I arrived at 7 a.m. and found only a sticky note on the table that read “This seat is taken, do not move!” Not even a book was there. At that moment, I really wanted to slap the note on the administrator’s face.
	b7 Declaration of power through material symbols	a7 iPad and laptop seating has become a new trend, students say “seating with valuable items has more deterrent power.”
	b8 Visualization occupancy	a8 I stuffed my review materials into a clear file bag and hung it on the back of the chair, declaring sovereignty without blocking the view, and it’s also not easy for the administrator to take.
	b9 Academic symbol usurpation	a9 There was a student who posted a “Experiment in Progress” A4 paper at the entrance of the discussion room, and no one went in for a week, but there was only an umbrella inside.
A1 Resource Scarcity Contradiction	b10 Time compression	a10 seat-taking is the tragedy of the commons: private cost vs. social cost; students commonly describe the time cost of finding a seat as high.
	b11 Seats are scarce	a11 The number of students N in class 11 exceeds the number of seats M in the library, resulting in scarce seating resources, which leads to seat hogging behavior.
	b12 Expansion trap	a12 Admission expansion led to a sharp decline in per-student resources, and even with the number of seats in a school in Inner Mongolia increasing from hundreds to 5,000, the supply still cannot meet the demand.
	b13 Outlet monopoly	a13 the person who occupies the seat wrapped the power outlet with tape, wrote “charging,” but the power strip was not actually powered on. [Source type: Social media; source ID: S-131-02.]
A13 Defensive Negotiation Behavior	b14 Negotiation period	a14 Both parties agree to divide the same seat with a “shared schedule,” morning A uses, afternoon B uses, conflicts are downgraded.
	b15 Strategic Negotiation Strategy	a15 replied: “When the admin came to collect the books, I rushed over and said ‘I need to go to the bathroom’.”
A5 Space Emotional Dependency	b16 Sentiment Dependency	a16 This window seat a16 is my home in the library; leaving it is impossible to concentrate on studying.
	b17 Spatial dependency	a17 Digital-library services may divert part of offline demand, but students still prefer the physical space for its “immersive learning atmosphere.”
	b18 Institutional dependence	a18 The learning community in the library is a loose combination of atomized individuals and small groups, yet it exhibits consistency in terms of rule constraints.
A6 Psychological Driving Factors	b19 Academic Panic	a19 postgraduate entrance exam has no seats, everyone is in a panic. There are people who work harder than me everywhere around.
	b20 Atmosphere infection	a20 Looked up, almost everyone was bending over in struggle. Studying in the library has a better atmosphere, and one would also be deeply infected by it.
	b21 Rigid demand for space under the examination economy	a21 Postgraduate Entrance Examination Declaration: “Having a fixed seat in the library is treated as essential for examination preparation.”
	b22 Involution Space Competition	a22 Occupying seats is a spatial manifestation of educational over-competition: the certificate economy (graduate entrance exams, civil service exams) gives rise to a battle for seats.
A3 Space Rights Conflict	b23 Functional conflict between management rights and property rights	a23 We are afraid when we patrol the library that students will take videos and say: “Infringement of private property.”
	b24 Common scene training	a24 (the poster): “Today, I put the books that were occupying seats directly at the lost and found desk, only to have them posted on the confession wall.” “What. Am I wrong?” 2nd floor: “You’ve violated someone’s ‘space sovereignty’, who else should we blame but you?” 3rd floor: “Is it reasonable to occupy public resources for private use? The person who posted the accusation probably has not studied property rights.
	b25 Anti-common ground tragedy	a25 The tragedy of the anti-commons theory reveals: occupying seats leads to the coexistence of effective supervision rights and inefficient usage rights, and resource waste originates from the fragmentation of property rights.
A14 Silent Resistance Strategy	b26 Person separation	a26 collects books every day, piled up like a small mountain, and students come to claim them like a lost and found.
	b27 Passive resistance	a27 Occupied seat items become “rubbish” after cleaning, students refuse to pick up and protest.
	b28 Herd psychology	a28 The herd mentality of taking and occupying seats is the core driving force of the seat-taking culture. Students admit, “Not taking a seat is a loss.”
A8 System Dynamic Practice	b29 Rule elasticity	a29 Many online comments support “If nothing is done within 30 min, it is considered to be giving up,” but the highest-upvoted reply is “People have three urgent needs; the system should not be too cold-blooded.”
	b30 Clearing rules	a30 Every evening after 21:30, the library will carry out centralized cleaning of items left behind, treating them as unclaimed. [Source type: Policy document; source ID: P-02-03.]
	b31 Rule countermeasure	a31 When clearing up items occupying seats, conflicts between students and librarians occur frequently, with abusive slogans posted on the door, reflecting the erosion of management authority. [Source type: Structured observation; source ID: O-19-05.]
A19 Space Functional Diversification	b32 Space over-claim	a32 library becomes a “temporary dormitory”: blankets and hand warmers occupy seats, reflecting students’ excessive demand for comfortable learning spaces.
	b33 Space substitution effect	a33 After installing multimedia in classroom 33, the school authorities closed the self-study room to prevent equipment damage, forcing students to flock to the library.
	b34 Space permutation	a34 textbook is present, that means I am here! My notebook backpack is here, it’s as if I am here.
A9 Disciplinary authority dissolution	b35 Space punishment	a35 Unbooked, items left for 30 min are considered to be occupying a seat, staff may clear the items and record one bad credit.
	b36 Hierarchical punishment	a36 Occupied items must be cleaned and stored in designated locations; students must obtain the signature of the college leader to claim them, and those who violate the rules 3 times consecutively will be reported to the department for handling.
	b37 Rigid flexibility imbalance	a37 An administrator was verbally abused by students for clearing up items occupying seats. After the library closed, the cleanup of items led to disputes over losses, leaving management in a dilemma of “strict management leads to conflicts, while laissez-faire leads to disorder.”
A20 Resource Dissipation Cycle	b38 Self-risk	a38 Readers are responsible for their own losses caused by occupying seats, and the library does not assume liability for compensation.
	b39 Collective and individual contradictions	a39 “The administrator always says ‘for all the students’ when cleaning the books, but who considers the emotional value of my 438 days of notes? The collective interest is a cold system of violence!”
	b40 Resource dissipation	a40 The “rent value dissipation” in the “occupy seat” of a40 manifests as: seat vacancy, queuing time loss, conflict costs, and the risk of item loss.
A16 Conspiracy exile structure	b41 Collaborative Spatial Production	a41 occupying seats is the result of “conspiracy” between the hierarchical organizational logic (school administration) and the collaborative logic (student spontaneous order).
	b42 Exile Ritual	a42 “When the administrator pushed the ‘exile car’ around the field to collect books, the entire field was as quiet as a execution ground—the moment my postgraduate exam notes were thrown into the ‘exile area,’ I felt like a spatial refugee”
A2 Class spatial acquisition	b43 Moral rationalization narrative	a43 Occupants of a seat often rationalize themselves with “striving for learning,” covering up their deprivation of others’ learning rights.
	b44 Structural rationality priority	a44 students propose “adding seats” as the preferred option, rather than moralizing, reflecting an understanding of structural contradictions.
	b45 Egoistic culture	a45 The intensified self-centeredness of only children leads to seat-occupying behavior, and the lack of sharing awareness is a deep moral deficiency.
A4 Hierarchical rights in space	b46 Stratified spatial convenience	a46 poor student relies more on free libraries due to being unable to afford off-campus self-study rooms, and unfair seat-taking exacerbates class segregation.
	b47 Spatial hierarchy order	a47 Students spontaneously formed a “seat-occupying culture”: VIP seats (by the window/near the power outlets) are occupied for extended periods, while seats at the edge have high turnover.
	b48 Usage privatization	a48 The seats in the library are non-exclusive public resources; occupying seats is actually a competitive behavior that transforms non-exclusive usage rights into temporary exclusivity.
A18 Alienation of spatial function	b49 Attribution Bias	a49 colleges attribute the occupation of seats to “student quality,” ignoring insufficient resource supply.
	b50 Powerlessness rights protection	a50 hates the despicable behavior of occupying seats, laments the lack of channels for rights protection. Students are forced to rush to occupy seats under the stars and the moon, which is a microcosm of unbalanced resource allocation.
A17 Space Monopoly	b51 Symbolized space guerrilla	a51 Students use strategies such as “taking seats with paper notes” and “one book taking multiple seats” to resist management, forming guerrilla-style seat-taking tactics.
	b52 Space violence	a52 Two girls got into a physical altercation over a seat, and one person’s thermal cup was smashed and deformed. [Source type: News media; source ID: N-07-02.]
	b53 Space monopoly	a53 Students tie the desks and chairs with iron chains, or use tape to flip and tie the chairs upside down on the desks, forming a “occupy the mountain and become a king” physical barrier.
A10 Technical training failure	b54 Technical rules overhead	a54 After reserving a seat at the venue, the table was piled high with books, and the reservation holder was forced to cancel and re-reserve—the seat hog declared “actual control” through items, undermining the legality of the reservation system. [Source type: Backend log; source ID: L-09-04.]
	b55 Technical training methods	a55 Scan and Reserve Seat System implements “Person-Code Binding,” short-term absence scans to reserve seats, long-term absence automatically releases, utilization reportedly increased.
	b56 Behavior trajectory prediction failure	a56 NetLogo simulation model shows: If the psychological distance demand intensity of students is moderate, the initial seat-finding failure rate remains high; a dynamic scheduling mechanism is needed to solve the “invalid flow.”
A11 Emotional Governance Failure	b57 Governance dilemma	a57 Management Paradox: Strict crackdown on seat occupation is resisted by students, and allowing it leads to resource waste. Need to balance efficiency and fairness.
	b58 Emotional venting	a58 Students vent their dissatisfaction through platforms like “Human Size Assistant,” but lack official feedback channels, leading to pent-up emotions turning into public anger on the internet.
A12 Digital Discipline Alienation	b59 Digital Discipline Diversification	a59 “The QR code reservation system requires ‘check-in and check-out’, students complain that ‘going to the bathroom to get water feels like being monitored by electronic shackles’, sacrificing technical rationality for humanized experience.”
	b60 Platform governance	a60 APP reservation + offline rental cabinet scanning + flexible use + credit score rewards and penalties.
A21 Psychological deprivation under imbalanced resource allocation	b61 Lack of fairness	a61 Some seat-holders report feeling guilty yet continue the behavior, forming cognitive dissonance.
	b62 Learned Helplessness	a62 Some students gave up the library due to repeated failure in finding seats, resulting in a decrease in learning efficiency.
A22 Moral alienation associated with rule failure	b63 Moral desensitization	a63 Moral desensitization: Under the influence of conformity, some students believe that occupying seats “does not violate morality.”
	b64 Space insecurity	a64 Always worried about the seat being taken, concentration decreases.

Source: Authors’ analysis.

**Table 3:** Axial coding table.

Main category	Initial category	Category intension
B1 Allocation conflicts associated with resource scarcity	A1 Resource Scarcity Contradiction	Limited physical seating and concentrated study demand are interpreted as contextual conditions that may be associated with seat-occupying behavior.
	A2 Class spatial acquisition	The materials suggest possible differences in spatial access associated with study purposes, social capital, and visiting time; these are interpreted as informal hierarchies within the corpus.
B2 Hierarchical structure under rights conflicts	A3 Space Rights Conflict	The conflict between students’ individual “usage” demands and the “sharing” nature of public resources is associated with disputes about the legitimacy of the “first-come, first-served” principle and the “reservation” system.
	A4 Hierarchical rights in space	Reservation arrangements and access privileges, such as designated examination-preparation areas, may be associated with unequal opportunities to use library space.
B3 Psychological and Emotional Spatial Dependency	A5 Space Emotional Dependency	Some accounts associate fixed seats with security, belonging, and a sense of private territory; these meanings may contribute to continued seat occupation.
	A6 Psychological Driving Factors	A preference for fixed seating, a sense of study ritual, and competitive anxiety are interpreted as possible psychological correlates of seat occupation.
B4 System intervention fails	A7 Space Order Game	The library exercises refined control over student behavior through rules (such as reservation systems and seat-time limits), reflecting the penetration of Foucault’s “disciplinary power” in space.
	A8 System Dynamic Practice	Reports describe a discrepancy between a formal prohibition on seat occupation and inconsistent enforcement, interpreted as a possible tension between stated rules and tacit acceptance.
	A9 Disciplinary authority dissolution	Accounts of conflicts over the clearing of personal items include doubts about the authority of rule enforcers; the direction of this relationship is not established.
B5 Space discipline failure	A10 Technical training failure	Complex reservation procedures and opportunities for circumvention, such as proxy reservations, are described as possible limitations of technological seat management.
	A11 Emotional Governance Failure	Accounts of poor implementation and limited student acceptance suggest possible concerns about the credibility and legitimacy of seat-reservation governance.
	A12 Digital Discipline Alienation	Reported evasion or misuse of digital management tools suggests that these tools may also be incorporated into informal seat-taking strategies.
B6 Resistance in spatial practice	A13 Defensive Negotiation Behavior	Students use items to occupy seats as a “non-verbal negotiation” with other potential users, establishing temporary boundaries of usage rights, and maintaining acquired spaces.
	A14 Silent Resistance Strategy	Some accounts connect reluctance to challenge occupied seats with anticipated trouble or limited access to complaint channels, alongside expressions of dissatisfaction on campus public accounts.
	A15 Symbolic Sovereignty Spatial Construction	Students practice micro-control over space through daily behaviors such as item reservation, oral negotiation, and eye intimidation.
	A16 Conspiracy exile structure	Repeated practices, such as early-morning queuing, are interpreted as possible signs of the normalization of seat occupation; collective unconscious habits were not directly assessed.
B7 Monopolistic internalization of power structure	A17 Space Monopoly	Some students monopolize high-quality space resources through means such as language threats, item occupation, and collaboration in small groups.
	A18 Alienation of spatial function	Reports of limited access to effective complaint channels coexist with expressions of unresolved dissatisfaction, suggesting a possible relationship between the two.
B8 Resource dissipation associated with spatial functional alienation	A19 Space Functional Diversification	Descriptions of books stored on seats or spaces claimed by small groups suggest a possible divergence between the intended learning function and everyday uses of library space.
	A20 Resource Dissipation Cycle	Seats occupied by belongings but not actively used may restrict access for other readers and contribute to ineffective use of space; effects on learning efficiency were not measured.
B9 Subject alienation under structural anomie	A21 Psychological deprivation under imbalanced resource allocation	Accounts of monopolistic or unequal access include expressions of unfairness and reduced autonomy; these are reported experiences rather than independently measured psychological effects.
	A22 Moral alienation associated with rule failure	Some accounts link inconsistent rule enforcement or informal rules with greater acceptance of seat occupation. This category interprets possible changes in moral evaluations without establishing their cause or a longitudinal psychological process.

Source: Authors’ analysis.

### 4.2 Axial coding and identification of main categories

Axial coding refines main categories from the initially separate concepts produced through open coding and examines relationships among them. It involves distinguishing the similarities and differences in the connotations of each category, establishing the attribution and associative relationships between categories, and then refining the main categories (Glaser and Strauss, 1967). This study, through repeated comparative analysis of the functional attributes, application scenarios, and internal logic of 22 initial categories, finally refines and integrates 9 main categories using axial-coding logic. For example, “the contradiction of resource scarcity” focuses on the objective limitations of library seats in terms of total quantity and distribution, while “class-based space acquisition” depicts the informal hierarchical phenomenon formed by students based on factors such as the order of visiting the library and social capital; through continuous comparison and analysis, the materials suggest an analytical relationship between the two: perceived or actual scarcity may create conditions under which hierarchical and competitive conflicts in access behavior become more likely. Based on this logical relationship, the two are integrated under the higher-dimensional theoretical concept of “distribution conflict,” thus forming the main category of “distribution conflict associated with resource scarcity.” This category integrates the coded phenomena and interprets seat occupation as one possible spatial manifestation of negotiated access and competition in the distribution of scarce public resources. The axial-coding results and the proposed relationships among categories are detailed in Tables 3, 4.

**Table 4:** Proposed relationships among categories.

Paradigm model component	Corresponding principal category	Typical manifestations
Condition	B1 resource scarcity contradiction	The materials describe difficulty obtaining seats during examination preparation and students queuing before library opening; these observations suggest a possible association with concentrated demand.
	B3 Psychological and Emotional Spatial Dependency	Students think that they can only learn if they have a fixed seat, and they have strong emotional attachment to the “old seat.”
Phenomenon	The Hierarchical Structure under B2 Right Conflict	Seats near windows and power outlets are repeatedly described as desirable (El-Khouly et al., 2026), and regular users may develop de facto priority claims.
Sequence of thought	Academic Involvement Pressure	Students generally believe that “studying hard in the library” is a necessary condition to cope with the fierce competition of postgraduate entrance examination and civil service examination.
	The Potential Injustice of Technological Governance	Some accounts associate complex reservation rules and opportunities for circumvention with concerns about unfair access.
Intervention study	Failure of B4 system intervention	Accounts describe inconsistent enforcement of the 30-min return rule alongside students’ attempts to retain access to seats.
	B5 space discipline failed	Accounts describe seat-clearing patrols alongside student resistance and less visible seat-occupation strategies.
Interaction strategy	Resistance in the B6 Space Practice	Using items to occupy seats, temporarily negotiating with others, or “renewing time” on the system when briefly leaving, etc.
	Monopolistic Internalization of B7 Right Structure	Some long-term seat-holders express a sense of ownership and describe small-group practices that may exclude other users.
Structure	B8 resource dissipation	The study room has empty seats with books, but the actual usage rate is low, and other students have no seats available.
	Subject Alienation under B9 Structural Disorder	Some accounts describe a shift from opposing seat occupation to participating in it. These reports suggest possible adaptation, without demonstrating within-person change over time.

Source: Authors’ analysis.

### 4.3 Selective coding

Selective coding refers to the systematic analysis of all the categories found to identify a core category, stringing all other categories together as a whole, and encompassing most research findings within a broader theoretical framework (Glaser and Strauss, 1967). Through continuous comparison, the social spatial structure of library seat occupation was identified as the core category linking the other categories. The relationships described below are interpretive links derived from the coded materials rather than causal effects established through site-level statistical testing. In the field of university libraries, perceived or actual resource scarcity is interpreted as a recurring condition associated with conflicts in seat allocation. Students’ psychological and emotional dependence on learning spaces, driven by psychological and emotional motivation, may interact with perceived resource scarcity and be associated with differences in access and rights claims. At the same time, reported difficulties in institutional rules and spatial discipline may be associated with student resistance and monopolistic rights claims. These practices are interpreted as potentially related to spatial functional alienation, informal spatial control, ineffective resource use, and reported adaptation. Such adaptation may, in turn, be associated with continued perceptions of scarcity and unequal access. These links organize the four layers of conditions, interventions, interactions, and feedback into a proposed interpretive process within the analyzed corpus; they do not establish a causal sequence or a self-reinforcing cycle over time.

### 4.4 Model construction

By encoding text materials related to occupying seats in university libraries, such as policies, news, social media, and physical materials, a multidimensional interpretive model of university-library seat occupation was developed around the internal logic of “condition-intervention-interaction-feedback.” The model interprets seat occupation as a situated practice that may reflect interactions among resources, designed settings, rules, behaviors, and psychological attachment within a specific spatial field. As shown in Figure 2.

**Figure 2:**
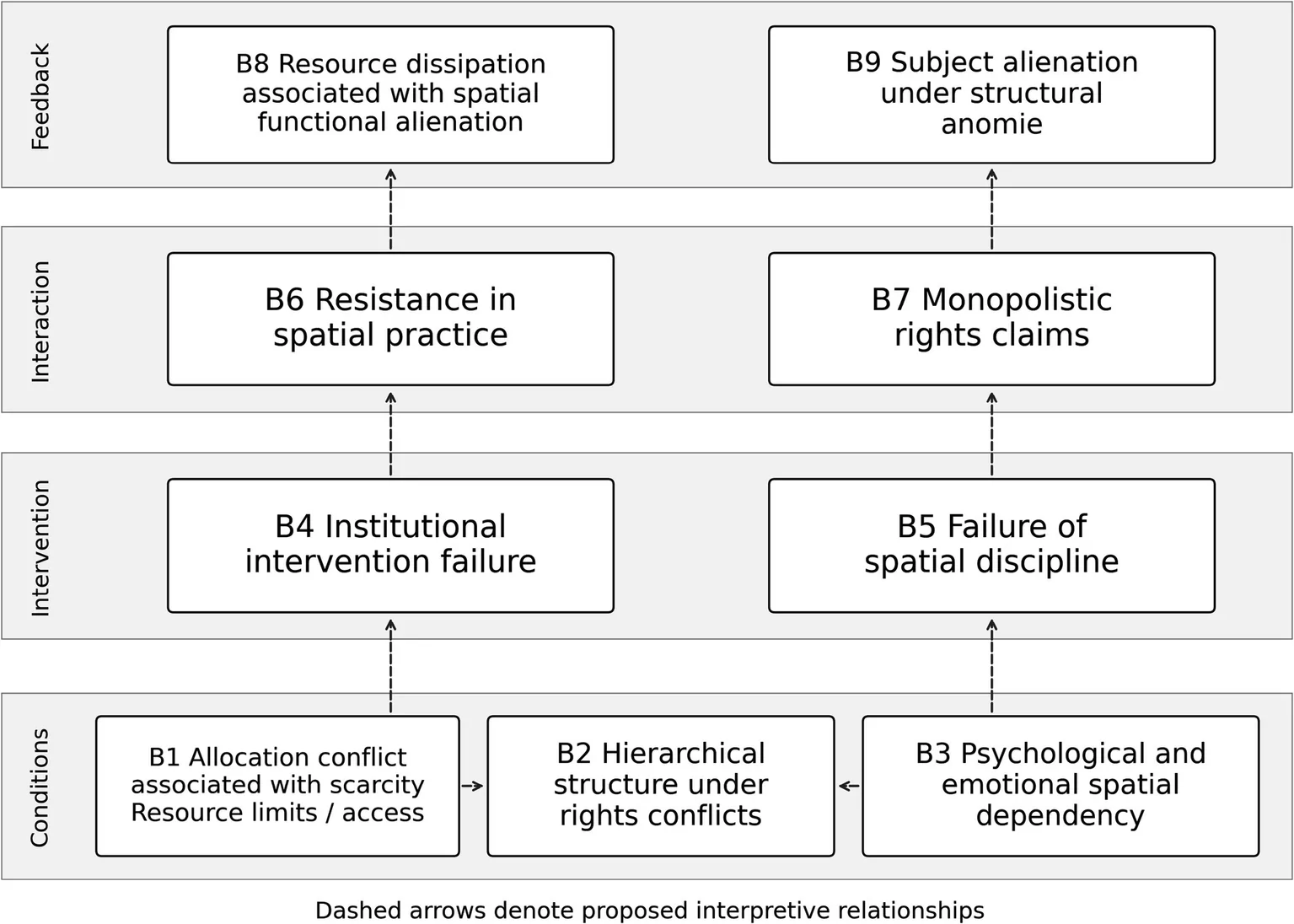
Interpretive model of the social spatial structure of university-library seat occupation (Source: Authors’ analysis). Dashed arrows indicate proposed interpretive relationships, not established causal effects or a verified temporal sequence.

### 4.5 Assessment of conceptual saturation within the retained corpus

Following constant comparison (Corbin and Strauss, 2015), the remaining 165 analyzable units were reviewed sequentially after the preliminary category system had been developed from the initial 331 units. Each unit was compared with the existing concepts, category properties, and proposed relationships using NVivo 14.0. No additional categories or relationships were identified in these 165 units within the retained corpus. Both sets of units came from the same predefined corpus; the subsequent assessment did not involve continued theoretical sampling or the collection of new sources in response to emerging categories. Two scholars in this field also independently reviewed the coding process and theoretical model. This review supported discussion of the interpretation but did not demonstrate theoretical saturation beyond the corpus. The finding is therefore limited to the absence of additional categories or relationships in the retained materials and does not establish theoretical completeness or generalizability to other settings.

## 5 Interpretation of the social-space model of university-library seat occupation

### 5.1 Structured tension in scarce space

The phenomenon of occupying seats in university libraries can be interpreted through the interplay of resource scarcity, seat desirability, and contested access. Resource scarcity is treated as a contextual starting point in the proposed interpretation. As a public resource, library seats have competitive consumption but are difficult to achieve effective exclusivity. Against the backdrop of increasing academic pressure, especially during the postgraduate and civil servant exam seasons, the demand for stable study spaces among students shows a rigid increase, while the supply of library seats is relatively fixed, which may be associated with a mismatch between supply and demand. Across the analyzed materials, examination periods were repeatedly associated with a mismatch between demand and available seats. This mismatch is treated as a recurring contextual condition rather than a sufficient cause of all subsequent conflicts. Unequal spatial access and allocation conflicts may be associated with perceived or actual resource scarcity. Where formal allocation is perceived as insufficient, accounts suggest that students may invoke informal “first-come, first-served” claims. By stacking textbooks, leaving personal belongings, and other methods, students try to establish a “temporary property” over specific seats. Observational data indicates that students assert space usage rights through item marking strategies, which may contribute to unequal access to particular seats, suggesting informal differences in access associated with the willingness or ability to maintain a claim. Psychological and emotional attachment to space may also be associated with these practices. Some accounts describe emotional attachment and habitual reliance on particular seats, interpreted as possible claims to “exclusive territories.” Social-media materials frequently used phrases such as “old position” and “exclusive seat” and linked fixed seats with perceived learning efficacy. These expressions suggest that particular seats may acquire territorial meaning and perceived legitimacy for users; psychological ownership and its effects were not directly measured. The proposed interpretation links resource scarcity, allocation conflict, unequal access, and emotional attachment as potentially related conditions. These links summarize patterns in the corpus rather than demonstrate how one condition produces another.

### 5.2 Structural failure of the governance system

The proposed intervention layer considers how institutional practices and technical governance difficulties may be related to the conditions described above. The categories “institutional intervention failure” and “spatial discipline failure” describe reported limitations of management measures and their possible relationships with ongoing allocation conflicts. Institutional intervention failure is manifested in rule ambiguity and weak enforcement. The management regulations of libraries often have elastic space, such as requiring “strict cleaning” while “avoiding conflicts,” which may create uncertainty for enforcers. Field observation logs suggested inconsistent handling of seat-occupation complaints and limited perceived deterrence from patrols. Across the coded materials, flexible enforcement and resistance to penalties were recurring themes, and some public comments described sanctions as a “paper tiger.” Such inconsistency may inadvertently normalize seat-occupying practices. Accounts of spatial discipline describe unresolved difficulties in technical management alongside concerns about unfair access. The seat reservation system was originally intended to optimize distribution, but complex procedures and reported instability may discourage use. More seriously, accounts of practices such as “technical cheating” include doubts about “algorithmic fairness.” When technical systems are difficult to use or can be circumvented, they may weaken the perceived authority of institutional rules. Within the interpretive model, the intervention layer connects contextual conditions with accounts of student interaction. Inconsistent enforcement and difficulties with technical systems may contribute to contested access and strategic responses. This is a proposed relationship; the corpus does not establish that these governance features activate previously latent conflicts or causally mediate subsequent behavior.

### 5.3 Competitive practices of spatial rights

The categories “Resistance in Spatial Practice” and “Monopolistic Rights Claims” can be interpreted as interacting access strategies. Resistance in spatial practice is the informal confrontation adopted by the disadvantaged party in resource acquisition. Accounts of limited formal channels coexist with descriptions of micro-strategies such as moving items and negotiating temporary use of seats. These accounts suggest a possible tension between dissatisfaction and reluctance to enter direct conflict, interpreted as a pattern of resistance and compromise. Monopolistic rights claims are the behaviors of the advantageous party in resource acquisition. Long-term placement of belongings and notes declaring “sovereignty” may be interpreted as claims to treat public seats as “quasi-private property.” In extreme cases, even market behaviors such as seat transfer or sale may occur, indicating a stronger informal privatization claim over space use. Accounts of monopolistic claims also describe dissatisfaction and resistance, suggesting a possible relationship between these practices. The interaction layer brings together reported strategies through which students negotiate access within these institutional and spatial settings. These strategies may be associated with challenges to institutional authority and unequal access. The materials suggest a possible divergence between formal access rules and informal spatial control, without establishing a fundamental transformation of rights over time. Figure 3 presents a conceptual framework derived from the grounded-theory analysis. The categories do not represent frequency distributions, and their arrangement does not indicate relative importance.

**Figure 3:**
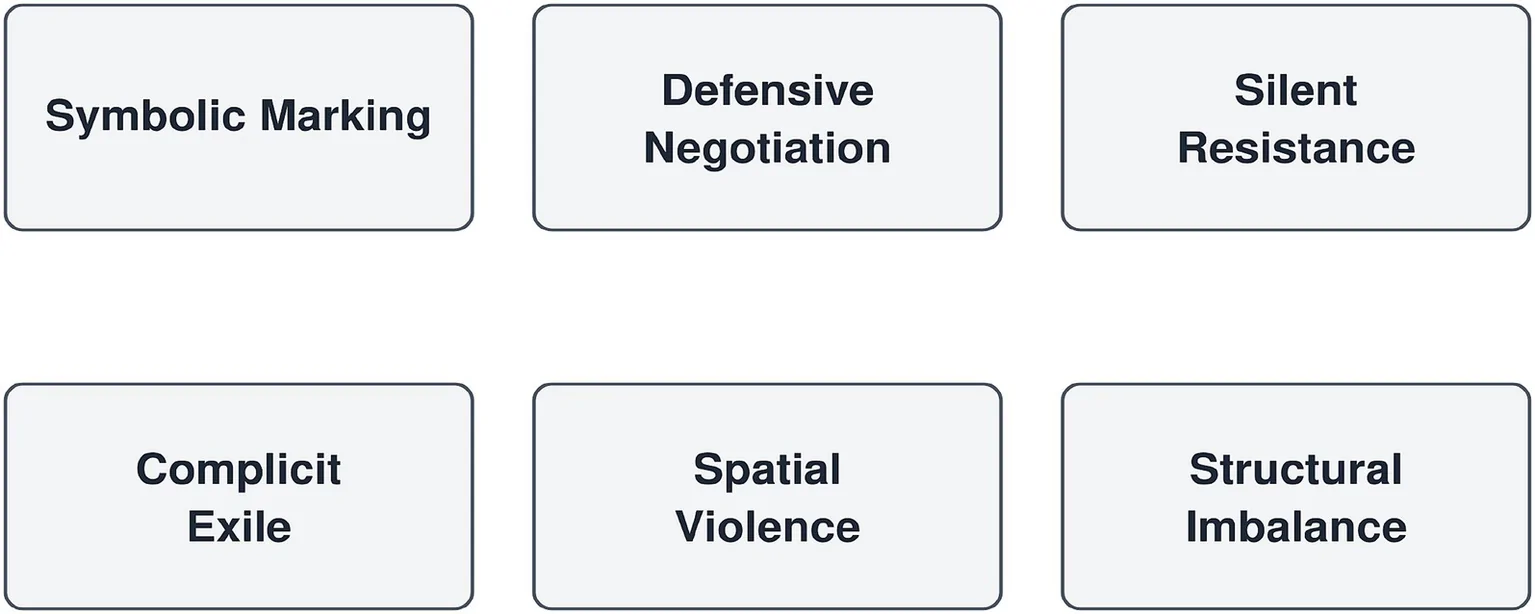
Conceptual framework of spatial-practice strategies (non-quantitative).

### 5.4 Proposed feedback relationships in spatial alienation

The feedback layer summarizes possible relationships between seat-occupying practices, ineffective resource use, and reported adaptation. These are interpretive links rather than demonstrated long-term consequences. This layer includes the categories “resource dissipation associated with spatial functional alienation” and “subject alienation under structural anomie.” The first concerns accounts in which seats intended for learning are used to store belongings or mark territory. Seats that are occupied by items but temporarily unused may restrict access for other readers and contribute to perceived scarcity; the corpus does not quantify the size or duration of this effect. The second concerns expressions of resignation, acceptance, or perceived legitimacy in accounts of seat occupation. Such expressions may indicate adaptation to informal practices, but they do not establish within-person psychological change or learning through observation over time. The model proposes that ineffective use of seating and acceptance of informal claims may be associated with the persistence of perceived scarcity and unequal access. Whether these relationships form a self-reinforcing process requires longitudinal investigation. Figure 4 depicts this proposed feedback structure rather than an empirically verified cycle.

**Figure 4:**
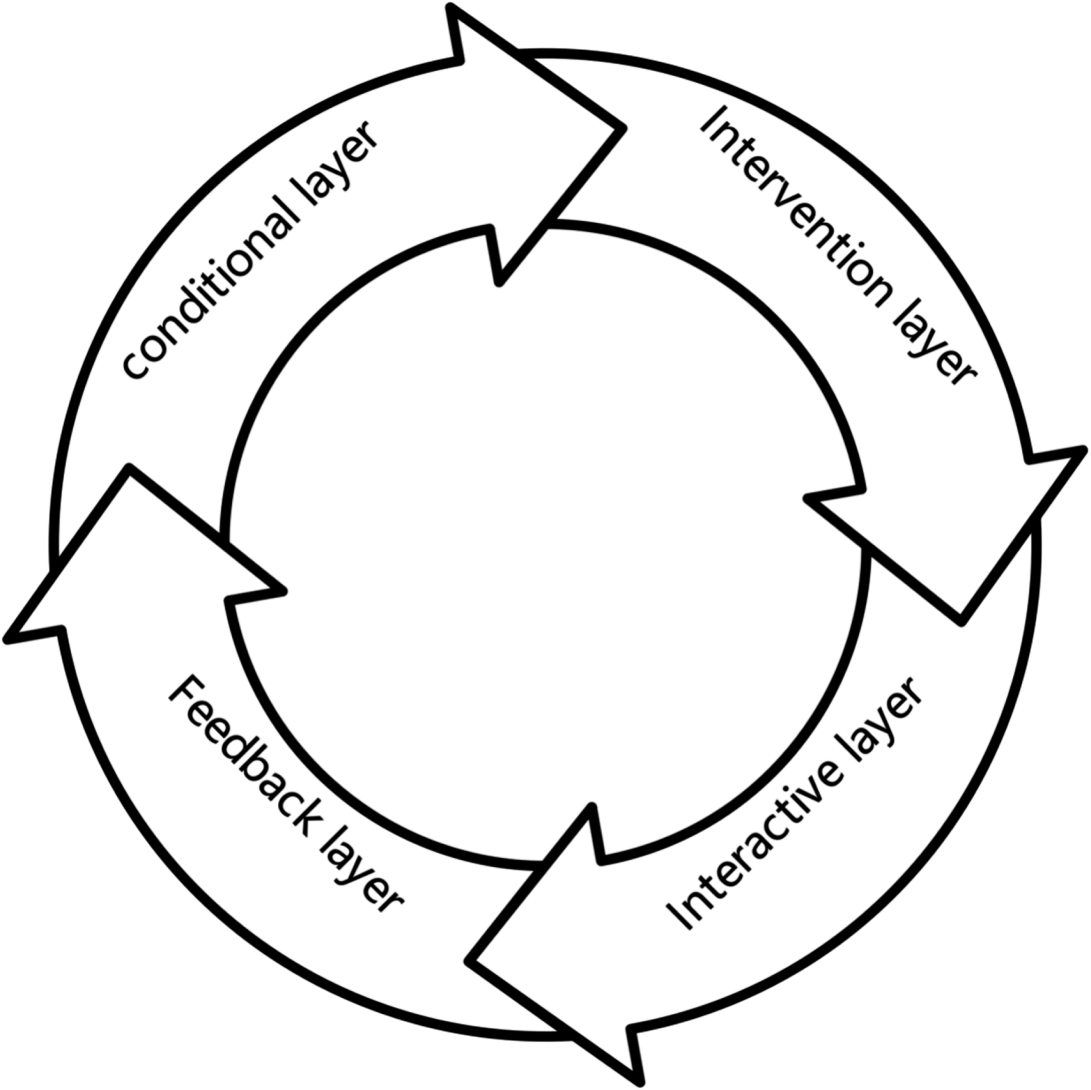
Proposed feedback loop (Source: Authors’ analysis). Arrows depict a proposed conceptual sequence and possible feedback; they do not demonstrate longitudinal change or causal effects.

Therefore, the “social spatial structure of university-library seat occupation” model constructed through grounded theory organizes proposed relationships from “structural conditions” to “institutional intervention,” then to “subject interaction,” and possible feedback. This model offers an interpretation in which seat occupation may emerge from interactions among resource allocation, designed spatial conditions, institutional practices, technological governance, individual behavior, and social psychology within particular settings. It is not only a management issue but also a profound social spatial issue. The framework suggests that management responses could consider several related conditions together; whether such responses alter the proposed feedback relationships remains to be evaluated.

## 6 Conclusion

### 6.1 Research findings

This study is based on the grounded theory method, conducting a systematic three-level coding analysis of the content of multi-source literature, public opinion materials, and empirical data, and deeply explores the issue of “the social spatial structure of university-library seat occupation.” The analysis identifies perceived or actual resource scarcity as a recurring condition that may intensify spatial conflicts during periods of concentrated demand. Limited seat availability may intensify distribution conflicts and contribute to informal differences in access among students. At the same time, the emotional dependence of students on the library as a “sacred place of learning” may be associated with their motivation to occupy seats. Inconsistent institutional intervention and difficulties in spatial governance may contribute to the normalization of seat-occupying practices. The corpus also describes strategic responses to management through the “one person occupying multiple seats” strategy, while some seat holders seek to maintain access through informal spatial control, alongside continued negotiation and conflict. Finally, seat occupation may contribute to ineffective resource use and psychological adaptation among student groups, potentially weakening perceived rule authority. Overall, the proposed model presents seat occupation as a situated process involving structural conditions, institutional and technological practices, student interaction, and possible feedback effects.

### 6.2 Theoretical contributions and practical insights

This study constructs a social spatial structure model of the occupation behavior in university libraries, not only providing an interpretive account of the possible formation process of the library seat occupation phenomenon, but also offering insights for the management practice of library spaces. The following sections discuss the theoretical contributions and practical implications.

#### 6.2.1 Theoretical contributions

The study’s theoretical contribution is to extend discussion of seat occupation from “conflict in space” toward an interpretive account of how space may be reproduced through everyday practice. The model suggests that seat occupation may reflect relationships among physical settings, management practices, and students’ everyday actions, providing an interpretive framework that extends beyond explanations based solely on morality or resource competition. At the level of theoretical dialogue, the study offers a conceptual extension of existing research. Library-design scholarship likewise treats spatial arrangement as a practical influence on use (Worpole, 2013), while the present analysis considers how users may renegotiate access through practices such as placing books on seats and taking turns to guard them. This not only deepens the understanding of the interaction between “space-behavior,” but also provides local empirical support for the theory of daily life practice, expanding the static spatial determinism to the dynamic theory of spatial production. Second, research conducted under changing institutional conditions shows that university-library activities remain sensitive to context (Kim and Yang, 2022). The present model therefore avoids a simple “rule-deviance” narrative. The model suggests a possible “negotiated order” associated with interactions such as tacit rules, temporary tacit consent, and selective enforcement between formal institutions and student practices. This finding shifts the analytical perspective from linear control and resistance to a relational understanding of how social space is reproduced in continuous negotiation, thereby enriching the theoretical discussion on how informal institutions are embedded and reshape formal structures.

#### 6.2.2 Practical insights

This study provides empirical insights for the spatial management and service optimization of university libraries based on a social spatial structure model. The proposed relationships suggest that reliance on administrative seat-clearing alone may have limitations; the effectiveness of alternative management strategies was not tested in this study. Managers should confront the structural contradiction of “distribution conflicts associated with resource scarcity” and start by optimizing the allocation of spatial resources, scientifically planning and laying out, and implementing a shared seating model with time slots and reservations to alleviate absolute scarcity. In response to the “hierarchicalization under rights conflicts” and the “internalization of the monopolistic nature of power structures,” weighted random or demand-priority allocation mechanisms could be evaluated as possible approaches to reducing informal monopolies and improving access. At the same time, managers could consider the meanings associated with “psychological and emotional spatial dependence” and guide students to form a rational spatial view through academic guidance and space publicity, while evaluating whether such measures affect attachment to fixed seats.

At the management level, facing the failure of “institutional intervention” and “spatial discipline,” managers could consider more responsive enforcement and graded warning procedures, with evaluation of their acceptability and effects. For “resistance in spatial practice,” it is advisable to dissipate and transform rather than suppress, and students can be absorbed as management volunteers, as a possible way to support collaborative management. Ultimately, to curb “resource dissipation associated with spatial functional alienation” and “subject alienation under structural anomie,” dynamic monitoring could inform allocation decisions and assessment of whether seats serve learning needs. These proposals require prospective evaluation.

Therefore, it is necessary for library space governance to shift from “technical control” to the consideration of “spatial justice,” starting with the design of flexible and inclusive spatial systems. Through multiple paths such as optimizing design, innovating systems, utilizing technology effectively, and promoting participation, libraries may be able to support collaboration in resource allocation; effects on seat conflicts, fairness, and efficiency remain to be tested.

#### 6.2.3 Limitations and future research

This study analyzed a predefined heterogeneous corpus; the absence of additional categories or relationships in the final 165 units does not establish that further theoretical sampling would yield no new insights. However, it should be pointed out that these materials themselves have certain limitations of generative perspective—whether it is the position of the issuing institution, the screening framework of the media, or the emotional tendency of a specific group of netizens, it is difficult to present the true motivations and complex interactions of all parties involved in the occupation behavior in a completely objective and neutral manner, which limits the comprehensiveness and objectivity of the theoretical interpretation. In addition, the source types differ in their units of analysis and evidentiary functions, and their integration depends partly on researcher judgment. Public online expressions do not disclose users’ private intentions, while field observations capture only visible behavior in three libraries and were not designed for site-level causal comparison. The coding process is therefore partly subjective, and the findings should not be generalized as prevalence estimates or universal mechanisms. The absence of detailed social-media retrieval and duplicate-handling records, site-specific observation schedules and recording procedures, and an itemized inventory of the 23 coded publications and 40 consulted publications limits reproducibility and source-level traceability. These gaps also constrain assessment of selection bias and differences between sites. Future studies should retain these records and use further theoretical sampling to explore categories beyond the present corpus. Based on this, subsequent research can be promoted from the following aspects: First, through the Delphi expert consultation method, the core path of “resource scarcity—power conflict—space function alienation” could be examined and refined in multiple rounds; second, cross-cultural comparisons are conducted to reveal the differences in students’ spatial cognition under different cultural backgrounds and their impact on spatial structure; in addition, it is also necessary to further expand the diversity of samples, promote the cross-application of various research methods, and try to carry out experimental evaluations of relevant spatial management policies, so as to provide more solid theoretical support for improving campus space fairness and promoting students’ wellbeing.

## Data Availability

The original contributions presented in the study are included in the article/supplementary material, further inquiries can be directed to the corresponding author.
